# Dynamic simulation of regulatory networks using SQUAD

**DOI:** 10.1186/1471-2105-8-462

**Published:** 2007-11-26

**Authors:** Alessandro Di Cara, Abhishek Garg, Giovanni De Micheli, Ioannis Xenarios, Luis Mendoza

**Affiliations:** 1 Merck Serono, Geneva, 9 Chemin des Mines, Switzerland; 2 EPFL, Lausanne, Building INF 341, Switzerland; 3 Swiss Institute of Bioinformatics, Vital-IT Group, Quartier Sorge – Batiment Genopode, CH-1015 Lausanne, Switzerland; 4 Instituto de Investigaciones Biomédicas, Universidad Nacional Autónoma de México, Ciudad Universitaria, CP04510, México

## Abstract

**Background:**

The ambition of most molecular biologists is the understanding of the intricate network of molecular interactions that control biological systems. As scientists uncover the components and the connectivity of these networks, it becomes possible to study their dynamical behavior as a whole and discover what is the specific role of each of their components. Since the behavior of a network is by no means intuitive, it becomes necessary to use computational models to understand its behavior and to be able to make predictions about it. Unfortunately, most current computational models describe small networks due to the scarcity of kinetic data available. To overcome this problem, we previously published a methodology to convert a signaling network into a dynamical system, even in the total absence of kinetic information. In this paper we present a software implementation of such methodology.

**Results:**

We developed SQUAD, a software for the dynamic simulation of signaling networks using the standardized qualitative dynamical systems approach. SQUAD converts the network into a discrete dynamical system, and it uses a binary decision diagram algorithm to identify all the steady states of the system. Then, the software creates a continuous dynamical system and localizes its steady states which are located near the steady states of the discrete system. The software permits to make simulations on the continuous system, allowing for the modification of several parameters. Importantly, SQUAD includes a framework for perturbing networks in a manner similar to what is performed in experimental laboratory protocols, for example by activating receptors or knocking out molecular components. Using this software we have been able to successfully reproduce the behavior of the regulatory network implicated in T-helper cell differentiation.

**Conclusion:**

The simulation of regulatory networks aims at predicting the behavior of a whole system when subject to stimuli, such as drugs, or determine the role of specific components within the network. The predictions can then be used to interpret and/or drive laboratory experiments. SQUAD provides a user-friendly graphical interface, accessible to both computational and experimental biologists for the fast qualitative simulation of large regulatory networks for which kinetic data is not necessarily available.

## Background

Over the last ten years molecular biologists and biochemists have shifted their focus from the study of single molecular entities to the study of mechanisms that govern molecular regulation and behavior within the cellular environment. Thanks to these efforts and the advent of high-throughput experimental technologies, researchers in the past have been able to dissect part of the intricate network of molecular interactions existing inside cells. Computational modeling of these regulatory networks aims at further understanding how their components are controlled, thus allowing the prediction of a set of non-obvious conclusions that can be subsequently addressed experimentally.

While data on the connectivity among molecules is becoming increasingly available, the stoichiometry and kinetic data of the biochemical reactions behind most of these connections remain to be elucidated. This knowledge gap is currently one of the major problems encountered by modelers, particularly when addressing the study of non-metabolic networks, such as signaling cascades. To tackle this issue, we have previously developed a methodology named *Standardized Qualitative Dynamical Systems *[1], which is a hybrid modeling method combining Boolean (discrete) and continuous modeling methodologies. This approach enables the dynamic simulation of regulatory networks in the absence of kinetic data.

Boolean networks have been used as a convenient modeling tool due to their computational simplicity, giving rise to a large body of literature regarding their suitability for modeling diverse biological processes (see [2-6] for some examples). In Boolean models, nodes are represented as variables that can attain only two values: 0 or 1, which represent the minimal and maximal state of activation, respectively. These nodes are connected through directed relationships, which can be either positive (*i.e. *activatory) or negative (*i.e. *inhibitory). In most cases, the specification of the network connectivity is not sufficient to determine the response of a given node to all its possible inputs. Because of this, modelers have to specify a Boolean function for every node, incorporating as much information related to the biological system as possible. While Boolean models give appropriate qualitative descriptions of the real biological systems, models can be refined by incorporating nodes with more than two levels of activation. This is usually possible when enough experimental evidence is available, allowing to distinguish among different levels of activation [5]. In other cases, however, defining multiple activation levels helps to incorporate distinct functional levels into the nodes [6]. Also, Boolean models have been extended by introducing stochasticity in the updating order of the nodes [7].

The standardized qualitative dynamical systems modeling approach can also be seen as an extension of the Boolean methodology, because it creates a system of ordinary differential equations (ODEs) that have a similar overall form to the step functions of Boolean models. In this way, the nodes in the network can attain a continuous range of values while at the same time allowing a direct comparison with Boolean nodes, since both implementations have their lowest value at 0, and their highest value at 1. This approach permits the qualitative modeling of networks, but allowing for the possibility of incorporating quantitative information into the model via the fitting of parameters.

In order to facilitate the use of standardized qualitative dynamical systems, we developed the SQUAD modeling suite, which provides a graphical interface accessible to modelers and biologists to perform simulations of regulatory or signaling networks. This tool can speed up the process of modeling signaling networks, because it provides a rapid way to obtain the set of all stable steady states of a network as implied by its topology. Also, it provides a convenient graphical user interface to make dynamical simulations and evaluate the effect of altering parameters.

Simulations using SQUAD are divided in three main parts. First, we supply the program with a directed graph representing the topology of a network, which can be done in the form of simple text or *sbml *file formats (see ahead). The program converts the network into a discrete dynamical system, and uses Boolean algorithms to identify all its stable steady states. Second, the program converts the network into a continuous dynamical system, in the form of a set of ODEs, and uses the steady states found in the discrete model as a guide to localize the stable steady states in the continuous model. And third, SQUAD allows the user to perform dynamic simulations, which may include perturbations, to assess the behavior of the network and identify the roles of specific nodes within the network. We describe the use of SQUAD, exemplifying it with simulations of the T-helper cell signaling pathway, which has been amply studied using different formalisms [1,8,9].

## Implementation

SQUAD is written in Java version 1.6. The network topology is loaded using any of three possible formats: *net*, *mml *and *sbml *(see Additional file 1 for a description). *Sbml *files are parsed using the jigcell SBML parser [10]. In order to represent the graphs both in memory and graphically, we use an extension to the JUNG library [11] built purposely to integrate parameters required for the dynamic simulations. The steady states of the loaded graphs are computed using a Reduced Order Binary Decision Diagram algorithm, described in detail in [8]. This algorithm is written in C++ and is integrated to the package through the Java Native Interface (JNI). The ordinary differential equations used for the dynamic simulations are implemented using the Open Source Physics framework (OSP) [12], using an adapted version of the Runge-Kutta4 solver to do the numerical computation. Furthermore, the OSP library is also used to display the activity using dynamic plots. The results of the simulations are stored in matrices using the dcolt package [13], which allows mapping of the matrices to a file system in order to reduce the memory expenditure. Finally, the user interface is built using java swing components, and the *synthetica *library is used to provide a consistent aspect on multiple platforms.

## Results

### Definition of the network topology

**Figure 1:**
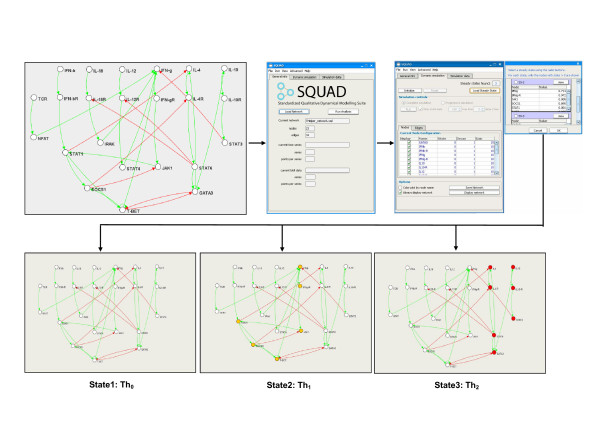
**Identification of steady states using SQUAD**. Graphical description of the identification of steady states using SQUAD. The model for the T-helper cell network (*upper left figure*) is loaded into SQUAD (*upper center figure*) using a CellDesigner *sbml *file. SQUAD displays the steady states identified in the network (*upper right figures*) through a scrollable list containing the values of the active nodes for each configuration. In addition the steady states can be visualized directly on the network topology (*lower figures*). For the T-helper network three steady states exist. Based on the molecular fingerprint, each state can be mapped to an existing biological state: State1 with all the nodes inactive corresponds to the Th0 cells. State2 with active IFN*γ *corresponds to the Th1 cells. State3 with active IL-4 corresponds to the Th2 state.

The first step towards modeling signaling networks is to define the components of the network and their connectivity. We symbolize the components of a network through nodes, represented as variables whose values reflect a state of activity. Nodes do not necessarily represent single molecules, but rather functional entities such as molecular complexes. In the T-helper network for example (Figure 1, upper left) the node describing the interferon-*γ *receptor (IFNg-R) represents a complex of multiple subunits that when active elicits the activation of the nodes downstream. The connectivity among nodes is expressed in terms of "activations" or "inhibitions". Once the topology of the network is established, it can be loaded into SQUAD to perform analyses and simulations.

SQUAD accepts three types of input formats: *net*, *mml *and *sbml *files. The *net *format is a simple text file, and the *mml *format is a XML file; both were developed specifically for SQUAD (see Additional file 1 for a description of the formats). Since defining a network topology for large networks in text format can be difficult and error-prone, we have included the possibility of using CellDesigner [14,15] generated files as input. CellDesigner is a free, widely used graphical tool that allows the easy construction and edition of diagrams of metabolic and regulatory networks. CellDesigner has an implementation of the SBML (Systems Biology Markup Language) format [16], used by a large number of modeling tools. Whenever CellDesigner files are used as input, SQUAD retains the spatial layout of the nodes providing a more intuitive interpretation of the simulation results (use Additional file 2 as an example). The distribution of SQUAD includes a folder containing the T-helper network sample files in the three aforementioned formats.

### Identification of network steady states using SQUAD

Biological systems are governed and regulated by intricate networks, most of the time containing feedback loops, whose activity is influenced by environmental stimuli. An illustrative example is the process of differentiation of precursor T-helper cells into effector Th1 or Th2 cells, in which a complex network controls the transition from a cell type to another according to the cytokines to which the precursor cells are exposed [17]. Dynamical systems may contain stable steady states, defined as specific activation states of the network, which do not change over time and are resistant to small perturbations. To find these stable steady states, it is necessary to translate the network topology into a dynamical system. We have previously published a methodology to automate this process [1]; hence, users of SQUAD only have to provide a network topology. The static representation of a network can be converted into a discrete dynamical system using the Equation 1 (described in [1]).

$$\begin{array}{c} x_{i}(t+1)=\{\begin{array}{cc} \left(x_{1}^{a}(t)\vee x_{2}^{a}(t)\ldots \vee x_{n}^{a}(t))\wedge \neg (x_{1}^{i}(t)\vee x_{2}^{i}(t)\ldots \vee x_{m}^{i}(t)\right) & a\\ x_{1}^{a}(t)\vee x_{2}^{a}(t)\ldots \vee x_{n}^{a}(t) & b\\ \neg (x_{1}^{i}(t)\vee x_{2}^{i}(t)\ldots \vee x_{m}^{i}(t)) & c \end{array}\\ \vee ,\wedge ,\text{and }\neg \text{ are the logical operators OR, AND, and NOT}\\ x_{i}\in \{0,1\}\\ \{x_{n}^{a}\}\text{ is the set of activators of }x_{i}\\ \{x_{n}^{i}\}\text{ is the set of inhibitors of }x_{i}\\ a\text{is used if }x_{i}\text{ has activators and inhibitors}\\ b\text{is used if }x_{i}\text{ has only activators}\\ c\text{is used if }x_{i}\text{ has only inhibitors} \end{array}$$

Once the network has been translated into a discrete dynamical system, it is possible to locate all its stable steady states using the Generalized Logical Analysis (GLA) [18], which is based on the analysis of the functionality of all the feedback loops that constitute the network. GLA is a well-established methodology to analyze the behavior of regulatory networks, even when they contain nodes with more than two levels of activation. However, the methodology has proved to scale badly for large networks. To address this issue, we implemented a Reduced Order Binary Decision Diagram (ROBDD) algorithm [8] in SQUAD. In contrast to GLA, the ROBDD algorithm works for networks containing only binary nodes.

ROBDD is a memory efficient data structure for representing the exponential state-space of logic functions, widely used in the field of electronic design automation and model checking. Using ROBDDs we can compute a set of subsequent network states in such a way that the state-space traversal can be performed very efficiently. Our implementation optimizes the use of ROBDDs by finding steady states without testing all the possible states of the network, allowing analysis of large regulatory networks. Using this algorithm we are able to identify the steady states of complex networks (>50 nodes) on a desktop computer in a matter of seconds (benchmarked on a Pentium4 2.1 GHz CPU). To support our claim, we provide a network of 111 nodes (Additional file 3) that can be tested for speed by the users. Another advantage of using the ROBDD algorithm is the identification of cyclic attractors, *i.e. *oscillating states. These states occur when the system does not reach a steady state, but rather a cycling pattern. We used SQUAD to identify the steady states of the T-helper cell network (Figure 1) either through GLA [1] or ROBDD. In both cases SQUAD identifies 3 stable steady states visible through the steady state selector panel (Figure 1, upper rightmost image), or on the graphical network layout (Figure 1, lower images). As previously reported [1], the steady states identified in the T-helper network can be mapped to a specific biological states of T-helper cells, namely the Th0, Th1 and Th2 cell types.

### Using SQUAD for studying the dynamic behavior of a network

SQUAD allows the identification of the stable steady states present in the network, but it does not provide information on the events that lead to these states. In other words, it finds the attractors but it does not give any information on the basins of attraction.

SQUAD automatically converts the static network into a continuous dynamical system (as explained in [1]) using Equation 2.

$$\begin{array}{c} \frac{dx_{i}}{dt}=\frac{-e^{0.5\text{h}}+e^{-h(\omega_{i})}}{\left(1-e^{0.5\text{h}})(1+e^{-h(\omega_{i}-0.5)}\right)}-\gamma_{i}x_{i}\\ \omega_{i}=\{\begin{array}{cc} \left(\frac{1-\sum \alpha_{n}}{\sum \alpha_{n}})(\frac{\sum \alpha_{n}x_{n}^{a}}{1+\sum \alpha_{n}x_{n}^{a}})(1-(\frac{1+\sum \beta_{m}}{\sum \beta_{m}})(\frac{\sum \beta_{m}x_{m}^{i}}{1+\sum \beta_{m}x_{m}^{i}})\right) & a\\ \left(\frac{1-\sum \alpha_{n}}{\sum \alpha_{n}})(\frac{\sum \alpha_{n}x_{n}^{a}}{1+\sum \alpha_{n}x_{n}^{a}}\right) & b\\ \left(1-(\frac{1+\sum \beta_{m}}{\sum \beta_{m}})(\frac{\sum \beta_{m}x_{m}^{i}}{1+\sum \beta_{m}x_{m}^{i}})\right) & c \end{array}\\ 0\leq x_{i}\leq 1\\ 0\leq \omega_{i}\leq 1\\ h,\alpha_{n},\beta_{m}>0\\ \gamma_{i}\geq 1\\ \{x_{n}^{a}\}\text{ is the set of activators of }x_{i}\\ \{x_{n}^{i}\}\text{ is the set of inhibitors of }x_{i}\\ a\text{is used if }x_{i}\text{ has activators and inhibitors}\\ b\text{is used if }x_{i}\text{ has only activators}\\ c\text{is used if }x_{i}\text{ has only inhibitors} \end{array}$$

Within this methodology, variables representing the activity of nodes are normalized, thus providing continuous levels of activation between 0 and 1 where, as in the discrete model, 1 represents the full-activation of a node.

In order to perform simulations, SQUAD solves numerically the continuous dynamical system starting from a given initial state, and a set of values for all parameters. By default, SQUAD sets all values of *α*'s (weight of activations), *β*'s (weight of inhibitions) to 1, and a value of 10 to *h *(the gain of the sigmoid). Users may change these values in the tables presenting the node and edge configurations. As for the initial states, the software uses the set of stable steady states found in the discrete implementation of the network. Here again, the user is able to specify any initial state that best matches the biological question addressed. In the case of the T-helper network for example, we can use the Th0 steady state, where all the nodes are set to 0, as a starting point for the dynamic simulations.

**Figure 2:**
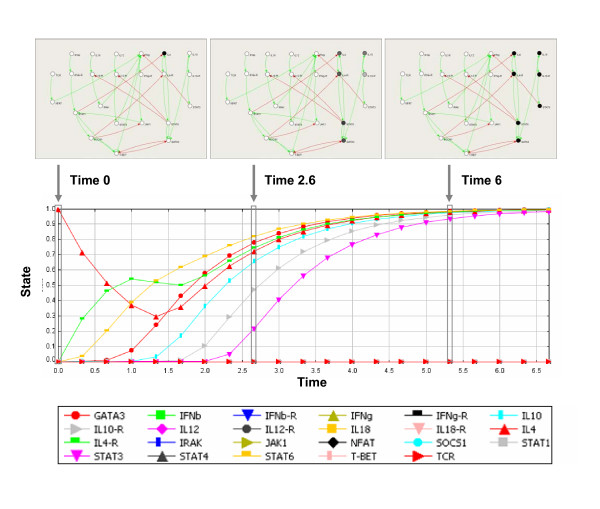
**Dynamic simulation of the T-helper regulatory network, in the presence of IL-4**. The plot provided by SQUAD displays the behavior of each component of the network according to time. The values range between 0 (inactive) and 1 (active). Time 0 indicates the time of addition of IL-4 to the network in the Th0 state. In addition to the plot display, SQUAD provides a dynamic network display (top panel) in which the nodes are colored in real-time according to the activation level from white (completely inactive) to black (completely active). The three top images are snapshots of the network at different time points. At Time 0 the network is in the Th0 and and a pulse of IL-4 is added to the system. The pulse originates a transitory state of activation (Time 2.6 is shown), which eventually leads the system to steady state representing the Th2 state (Time 6 and onwards). Time is expressed in arbitrary units.

SQUAD provides a number of graphical utilities to perform simulations. In the steady state selector panel it is possible to choose the starting state from the list of stable steady states automatically found by the system. The selected state can then be further modified to simulate alternative initial states, allowing for the inclusion of perturbations. The results of the simulations are shown by a plot of the activity of each node against time (Figure 2). Importantly, since the equations used for the dynamic simulation are not fitted with experimentally determined kinetic values, the time is expressed in arbitrary units.

Simulations in SQUAD can be performed in two modes. The *complete run *option sets the dynamic simulation to stop either at a pre-defined time point, or when a steady state is reached. By contrast, in the *progressive simulation *mode the user is able to control the speed of the simulation and stop it at will. In addition to the plot of time-series, SQUAD also displays the activation status of all nodes. This is particularly useful to make a more intuitive display of how some signals propagate through the network. To exemplify the use of these graphical tools, Figure 2 presents a simulation of the behavior of the Th0 steady state in which the IL-4 node is activated, thus mimicking the addition of IL-4 ligand to the network. We observe that the network moves to the Th2 steady state in response to the IL-4 ligand.

The algorithm behind SQUAD has already been shown to correctly describe the qualitative behavior of a large regulatory network [1]. Different networks, however, might require special manipulations before being analyzed by SQUAD. This is the case whenever there are nodes implementing the *AND *logical function. Equation 1 indicates that a node integrates the total input by means of *OR *functions, apparently hindering the use of *AND *relationships between input nodes. This seeming problem can be solved by the introduction of an intermediary node. Suppose the user needs to include a node *X *that becomes active only when nodes *A *and *B *are both active. In this case, it is necessary to decompose one of the direct activations, say from *A *to *X*, into a pair of inhibitions via an extra node, *C*, created *ad hoc*. The final topology would then become *A*¬*C*¬*X *← *B*. By applying Equation 1 we can see that *X *= *B *∧ ¬ *C*, but since *C *= ¬ *A *then *X *= *A *∧ *B*, which is the desired *AND *relationship between the *A *and *B *nodes over *X*. This solution for including AND gates increases the number of nodes in the network, and thus it might introduce some extra states in the attractors. Hence, the user has to be careful to eliminate from the final list of attractor the states of these intermediate nodes. Nevertheless, we are currently working on the explicit incorporation of *AND *gates into our methodology.

### Using laboratory protocols for modeling

As discussed in the previous section, dynamic simulations are extremely useful for assessing how a network behaves in response to different stimuli. In the work described so far we have addressed the influence of stimuli such as IL-4 on the initial steady state. Biological experimental protocols however often rely on perturbations using multiple stimuli, at different times and for varying durations. To map the computational simulations to such biological experiments we have included in SQUAD a framework for performing dynamic perturbations.

**Figure 3:**
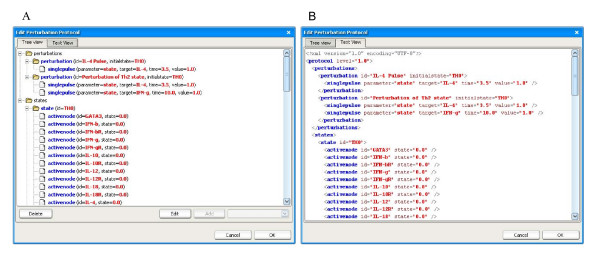
**Screenshots of the perturbation protocol designer tool**. This tool is used to create and edit perturbation protocols. The protocol contains two perturbations. For each perturbation an initial state is described. Each perturbation has one ore more "action" elements, which specify the types of perturbations to be performed. More complex protocols can include other types of actions such as perturbations over a time range or node knockouts. (A) Tree view: contains wizard buttons to add, edit and delete parts of the protocol. (B) Text view: provides a textual representation of the protocol file. Once generated, the protocol file can be saved and re-used.

The perturbations to be performed are listed within a protocol file written in a dedicated XML format (see Additional file 4, with extension *prt*). The file describes a set of initial network states and a set of perturbations. Each perturbation corresponds to a separate experiment containing an initial state and a set of actions specifying the node(s) to perturb, the perturbation value(s) as well as the duration and timing of the perturbations. Different types of actions can be specified. For example the *singlepulse *action modifies the node at a single time point, while *rangepulse *maintains the perturbation for a determined time period. The protocol file can be created within SQUAD using a wizard tool, which ensures that the protocol file has a valid format (Figure 3). Using these protocols it is possible to reproduce existing biological experiments computationally, or to test new experimental designs. Furthermore, having a file format to specify dynamic simulations allows for the storing, exchanging and comparisons of protocols.

**Figure 4:**
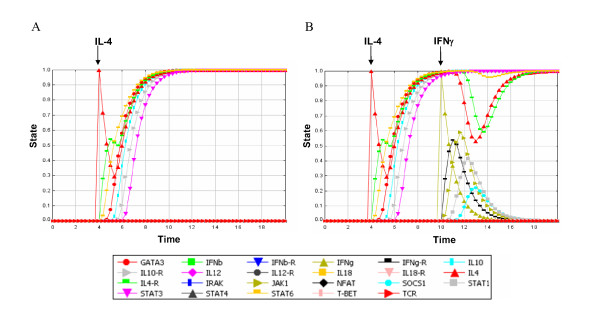
**Perturbations of the Th0 state with IL-4 and IFN-*γ***. Each curve represents the activity of a node in function of time. (A) Effect of IL-4 (red upward triangle) on the Th0 state (B) effect of IL-4 (red upward triangle) on the Th0 state and effect of IFN*γ *(yellow upward triangle) on the IL-4 induced Th2 state.

We have used the perturbation framework on the T-helper cell network to assess the effects of IL-4 and IFN-*γ *(Figure 4 and Additional file 4). As shown in the previous section, the addition of IL-4 to the Th0 state moves the network towards the Th2 steady state (Figure 4A). Similarly, using the perturbation protocols we tested the effect of adding an IFN-*γ *pulse on the Th2 steady state (Figure 4B). Under these circumstances the Th2 state is temporarily perturbed, but returns to the Th2 state, consistent with experimental data showing the stability of Th2 cells [19].

## Conclusion

Here, we demonstrate how the use of SQUAD helps to simulate the behavior of regulatory networks, modeled with the standardized qualitative dynamical systems methodology. This method can be used to study signaling or regulatory networks where there is little or no kinetic data available, but there is a good knowledge of the network topology. While there are no restrictions in the kind of biological phenomena that can be modeled with our methodology, it seems to be particularly suitable for differentiation processes, where multiple stable steady states can be interpreted as different cell types, as we have shown for the Th model [1]. SQUAD automatically locates the alternative stable steady states of the system, and the user can gain further knowledge of the model by using the several simulation tools provided, specially the ability to perform perturbations, mutations, and changes in the parameters to observe the effect on the nature and number of attractors.

The methodology implemented by SQUAD is able to find the attractors of the network under study. This information is relevant because it permits the user to know which are the alternative asymptotic behaviors that can be reached by the system. In its current implementation SQUAD does not provide information regarding the basins of attraction. That is, the software does not provide the user with a list of all the possible states that can reach a given steady state. However, we have introduced the possibility to export the model to a file in GNU Octave format [20], which can be modified to accomplish this job. The user has to be aware that the dynamical system exported by SQUAD is the one obtained by applying Equation 2, which is a deterministic set of ordinary differential equations.

The algorithms behind SQUAD have been thoroughly tested for the location of fixed-point attractors. Nevertheless, the methodology used by SQUAD can also identify cyclic attractors (see [8] for details). If a cyclic attractor is found, SQUAD displays one of its transitory states which, if used as an initial state in the dynamic simulations, will resume the oscillatory behavior of the system. The software, however, does not provide an automated way to visualize all possible cycles in the network when it is modeled as a discrete dynamical state, as GINSim does [3].

With SQUAD we have extended the original methodology published in [1] to allow the analysis of large regulatory networks (>50 nodes) by implementing a Reduced Order Binary Decision Diagram algorithm. In addition we have streamlined the whole modeling process by developing SQUAD with an intuitive interface that provides a dynamic visualization of the network. Many modeling software packages rely on their own input format. While this is the case for SQUAD, the program is also able to read CellDesigner *sbml *files [15], a file format widely used in the modeling community providing a seamless integration with several network drawing software and model repositories [21].

SQUAD is aimed at helping the scientific community to understand the global qualitative dynamical behavior of signaling networks. First, people may use the software to gain insight on the dynamical implications of a given network topology. It is often the case that there is enough information about the topology of a particular network, but there is no information on the biochemical reactions behind it. In this case, SQUAD can be of help by adding a dynamical dimension to such topologies, by creating dynamical systems based only on the network architecture. Experimental biologists may be specially benefited by testing different topologies and deciding which one offers the most accurate dynamical description to their phenomena of interest. Users with a moderate knowledge in modeling can benefit of the user-friendly interface that allows to modify the initial states, strength of interactions, decay rates, perturbation protocols and plotting capabilities of SQUAD. All these capabilities will help the user in predicting the dynamic behavior of a network in response to multiple stimuli or mutations, being knockouts, over expression, or a mixture thereof, as well as pinpointing the roles of specific components within a network.

There are other software packages that are used to generate qualitative models of regulatory networks, notably GINSim [3,22], Genetic Network Analyzer (GNA) [23] and CellNetAnalyzer (CNA) [24]. These modeling suites simulate networks implemented as dynamical systems, using either discrete (GINSim, CNA) or continuous systems (GNA). These packages have been shown to qualitatively reproduce the activation patterns of experimentally validated regulatory networks. For example, GNA has been used to analyze the network underlying the initiation of sporulation in *Bacillus subtilis *[25], GINSim to study the formation of discrete expression region of gap-genes *Drosophila *[26], and CellNetAnalyzer in T-Cell activation [27]. SQUAD complements these approaches by allowing the qualitative modeling using a set of non-linear ordinary differential equations. With the increase of published signaling networks, it will be possible in the future to realize a benchmark among these software packages to compare their strengths and weaknesses. For doing that, however, it would be very useful to develop a common file format. For the time being, SQUAD and CellNetAnalyzer have the possibility to read *sbml *files, which is becoming a widely used standard in the modeling community.

SQUAD has been implemented as part of the ENFIN project [28,29] committed to provide an integration of computational approaches in systems biology and the development of tools for harnessing biological system-level data. SQUAD constitutes an easy-to-use regulatory network modeling tool, accessible to both computational and experimental biologists.

## Availability and requirements

SQUAD can be downloaded from [30]. The website contains a quick-start guide and a detailed tutorial. Also, a copy of the SQUAD executables is provided in Additional file 5.

**Operating system(s)**: Windows and Linux (32-bit versions).

**Requirements**: Java 1.6 or higher.

**Programming language**: Java and C++ for the ROBDD algorithm.

**Restrictions of use by non-academic users**: None.

## Authors' contributions

AD and LM wrote the code for SQUAD. AG wrote the ROBDD algorithm. IX and GD supervised and coordinated the project. All authors have read and approved the final manuscript.

## Supplementary Material

Additional file 1 Description of SQUAD input formats. Description of the file formats used for loading network topology data into SQUAD. Click here for file

Additional file 2 T-helper regulatory network in CellDesigner SBML format. Representation of the T-helper regulatory network using the CellDesigner SBML format. The file can be viewed using CellDesigner [15] and imported directly into SQUAD for the analysis. Click here for file

Additional file 3 Network file. A sample network file containing a network of 111 nodes and 116 edges. Click here for file

Additional file 4 Perturbation protocol. Example of perturbation protocol file used to perform the simulations. The file describes two perturbations. The first one on the Th0 state with IL-4, the second on the Th0 state with IL-4 and subsequent IFN-*γ *addition. The file can be loaded from the Perturbation tool in SQUAD. The file also contains three different perturbations of the IL-18 node aimed at demonstrating the use of the different pulse types: "constant", "range" and "single". Click here for file

Additional file 5 SQUAD software. Compressed Zip file containing the binaries of SQUAD. To run SQUAD: Unzip the folder, in linux type *chmod +x Squad2 *and run it with *./Squad*, or in windows click on *Squad2.bat*. A quick-start guide and a more detailed tutorial can be found on the SQUAD home page [30]. The zip file encloses a samples folder containing all the files described in this manuscript. Click here for file
