# Supplementary material for: Dynamic simulation of regulatory networks using SQUAD
Source: BMC Bioinformatics. 2007 Nov 26;8:462. doi: 10.1186/1471-2105-8-462 (PMC2238325; doi:10.1186/1471-2105-8-462)

Additional File 1

File formats used by SQUAD

The first step towards modeling signaling networks is to define the components of the network and their connectivity. We symbolize the components of a network through nodes, represented as variables whose values reflect a state of activity. Nodes do not necessarily represent single molecules, but rather functional entities such as molecular complexes. The connectivity among nodes is expressed in terms of "activations" or "inhibitions". Once the topology of the network is established, it can be loaded into SQUAD to perform analyses and simulations. SQUAD accepts three types of input formats: *net*, *mml* and *sbml* files.

NET format

Net files offer the easiest input format because it is a plain text and human-readable format that describes an interaction between two nodes per line. Each interaction can be either positive (->) or negative (-|). For example:

```
GATA3 -> GATA3
STAT6 -> GATA3
T-bet -| GATA3
IFN-b -> IFN-bR
IRAK -> IFN-g
NFAT -> IFN-g
GATA3 -| T-bet
STAT1 -> T-bet
T-bet -> T-bet
```

MML format

A much richer file format, *mml*, is a plain-text computer-readable format that allows the specification not only of the topology of the network, but also of some supplementary information that might be needed for the dynamical modeling, such as the decay rate of the nodes and the strength of the reactions, but also the position of the nodes in the display and the steady states of the network. In addition, the *mml* file format is used to save the analysis data generated by SQUAD, avoiding the need to re-analyze the same network multiple times. An example of *mml* is shown below.

```
<?xml version="1.0" encoding="UTF-8"?>
<mml level="2">
  <!--Modelomics Markup Language (MML) v2.0-->
  <model />
  <graph>
    <nodes>
      <node id="GATA3" state="0" decay="1" gain="10" visible="true" />
      <node id="IFN-b" state="0" decay="1" gain="10" visible="true" />
      <node id="IFN-bR" state="0" decay="1" gain="10" visible="true" />
      <node id="IFN-g" state="0" decay="1" gain="10" visible="true" />
```

```
    <node id="IFN-gR" state="0" decay="1" gain="10" visible="true" />
    <node id="IL-10" state="0" decay="1" gain="10" visible="true" />
    <node id="IL-10R" state="0" decay="1" gain="10" visible="true" />
    <node id="IL-12" state="0" decay="1" gain="10" visible="true" />
    <node id="IL-12R" state="0" decay="1" gain="10" visible="true" />
    <node id="IL-18" state="0" decay="1" gain="10" visible="true" />
    <node id="IL-18R" state="0" decay="1" gain="10" visible="true" />
    <node id="IL-4" state="0" decay="1" gain="10" visible="true" />
    <node id="IL-4R" state="0" decay="1" gain="10" visible="true" />
    <node id="IRAK" state="0" decay="1" gain="10" visible="true" />
    <node id="JAK1" state="0" decay="1" gain="10" visible="true" />
    <node id="NFAT" state="0" decay="1" gain="10" visible="true" />
    <node id="SOCS1" state="0" decay="1" gain="10" visible="true" />
    <node id="STAT1" state="0" decay="1" gain="10" visible="true" />
    <node id="STAT3" state="0" decay="1" gain="10" visible="true" />
    <node id="STAT4" state="0" decay="1" gain="10" visible="true" />
    <node id="STAT6" state="0" decay="1" gain="10" visible="true" />
    <node id="T-bet" state="0" decay="1" gain="10" visible="true" />
    <node id="TCR" state="0" decay="1" gain="10" visible="true" />
   </nodes>
   <edges>
    <edge id="GATA3 -- GATA3" source="GATA3" target="GATA3" sign="positive" weight="1" />
    <edge id="STAT1 -- SOCS1" source="STAT1" target="SOCS1" sign="positive" weight="1" />
    <edge id="IL-18 -- IL-18R" source="IL-18" target="IL-18R" sign="positive" weight="1" />
    <edge id="SOCS1 -| IL-4R" source="SOCS1" target="IL-4R" sign="negative" weight="1" />
    <edge id="STAT4 -- IFN-g" source="STAT4" target="IFN-g" sign="positive" weight="1" />
    <edge id="IL-4 -- IL-4R" source="IL-4" target="IL-4R" sign="positive" weight="1" />
    <edge id="T-bet -| GATA3" source="T-bet" target="GATA3" sign="negative" weight="1" />
    <edge id="IL-12 -- IL-12R" source="IL-12" target="IL-12R" sign="positive" weight="1" />
    <edge id="IRAK -- IFN-g" source="IRAK" target="IFN-g" sign="positive" weight="1" />
    <edge id="GATA3 -- IL-10" source="GATA3" target="IL-10" sign="positive" weight="1" />
    <edge id="GATA3 -| T-bet" source="GATA3" target="T-bet" sign="negative" weight="1" />
    <edge id="STAT6 -| IL-12R" source="STAT6" target="IL-12R" sign="negative" weight="1" />
    <edge id="IL-18R -- IRAK" source="IL-18R" target="IRAK" sign="positive" weight="1" />
    <edge id="IL-4R -- STAT6" source="IL-4R" target="STAT6" sign="positive" weight="1" />
    <edge id="JAK1 -- STAT1" source="JAK1" target="STAT1" sign="positive" weight="1" />
    <edge id="GATA3 -| STAT4" source="GATA3" target="STAT4" sign="negative" weight="1" />
    <edge id="IL-12R -- STAT4" source="IL-12R" target="STAT4" sign="positive" weight="1" />
    <edge id="IL-10 -- IL-10R" source="IL-10" target="IL-10R" sign="positive" weight="1" />
    <edge id="SOCS1 -| JAK1" source="SOCS1" target="JAK1" sign="negative" weight="1" />
    <edge id="TCR -- NFAT" source="TCR" target="NFAT" sign="positive" weight="1" />
    <edge id="IFN-b -- IFN-bR" source="IFN-b" target="IFN-bR" sign="positive" weight="1" />
    <edge id="STAT1 -| IL-4" source="STAT1" target="IL-4" sign="negative" weight="1" />
    <edge id="IL-10R -- STAT3" source="IL-10R" target="STAT3" sign="positive" weight="1" />
    <edge id="T-bet -- IFN-g" source="T-bet" target="IFN-g" sign="positive" weight="1" />
    <edge id="GATA3 -- IL-4" source="GATA3" target="IL-4" sign="positive" weight="1" />
    <edge id="STAT3 -| IFN-g" source="STAT3" target="IFN-g" sign="negative" weight="1" />
    <edge id="IFN-g -- IFN-gR" source="IFN-g" target="IFN-gR" sign="positive" weight="1" />
    <edge id="T-bet -- SOCS1" source="T-bet" target="SOCS1" sign="positive" weight="1" />
    <edge id="NFAT -- IFN-g" source="NFAT" target="IFN-g" sign="positive" weight="1" />
    <edge id="IFN-gR -- JAK1" source="IFN-gR" target="JAK1" sign="positive" weight="1" />
    <edge id="STAT6 -- GATA3" source="STAT6" target="GATA3" sign="positive" weight="1" />
    <edge id="STAT1 -- T-bet" source="STAT1" target="T-bet" sign="positive" weight="1" />
    <edge id="T-bet -- T-bet" source="T-bet" target="T-bet" sign="positive" weight="1" />
    <edge id="STAT6 -| IL-18R" source="STAT6" target="IL-18R" sign="negative" weight="1" />
    <edge id="IFN-bR -- STAT1" source="IFN-bR" target="STAT1" sign="positive" weight="1" />
   </edges>
   <states />
  </graph>
 </mml>
```


SBML format

Since defining a network topology in text format can be difficult and error-prone for large networks, we have included the possibility of using CellDesigner (http://celldesigner.org) generated files as input. CellDesigner is a free, widely used graphical tool that allows to

easily construct and edit network diagrams. CellDesigner has an implementation of the SBML (systems biology meta language) format used by a large number of modeling tools. Whenever CellDesigner files are used as input, SQUAD retains the spatial layout of the nodes providing a more intuitive interpretation of the simulation results. Below is a screenshot of the T-Helper network represented in CellDesigner.

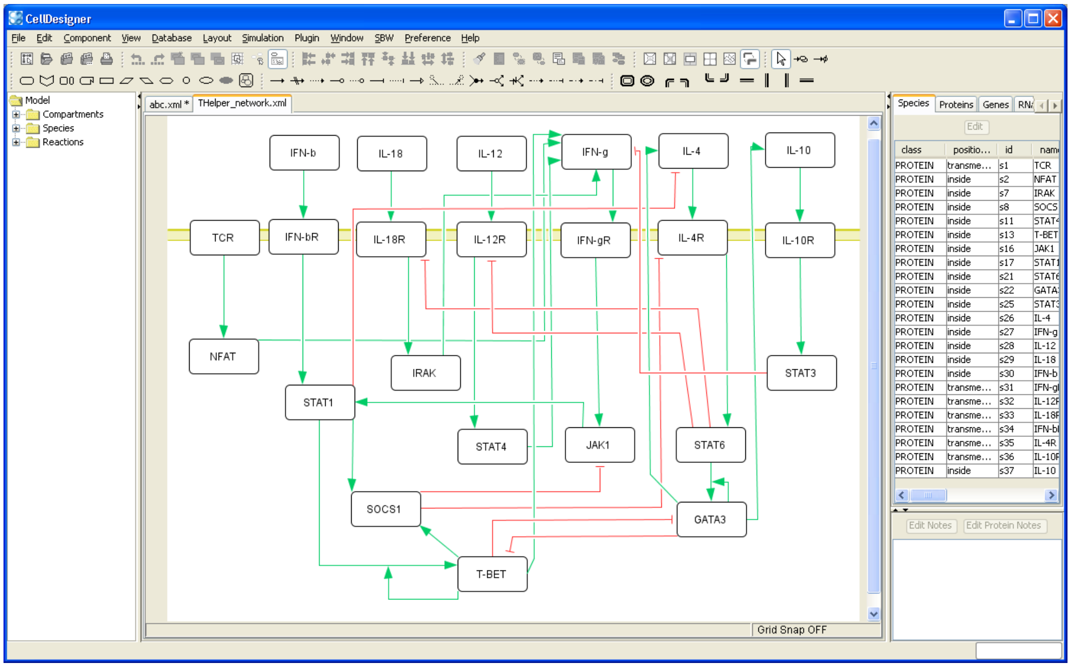

Supplement: Additional file 1 — Description of SQUAD input formats. Description of the file formats used for loading network topology data into SQUAD. [file 1471-2105-8-462-S1.pdf]
